# Bimodal Transformer with Regional EEG Data for Accurate Gameplay Regularity Classification

**DOI:** 10.3390/brainsci14030282

**Published:** 2024-03-15

**Authors:** Jinui Lee, Jae-Ho Han

**Affiliations:** 1 Department of Brain and Cognitive Engineering, Korea University, 145 Anam Rd., Seoul 02841, Republic of Korea; jinui@korea.ac.kr; 2 Interdisciplinary Program in Precision Public Health, Korea University, 145 Anam Rd., Seoul 02841, Republic of Korea; 3 Department of Artificial Intelligence, Korea University, 145 Anam Rd., Seoul 02841, Republic of Korea

**Keywords:** deep learning, electroencephalography, gaming industry, classification, Transformer

## Abstract

As games have been applied across various fields, including education and healthcare, numerous new games tailored to each field have emerged. Therefore, understanding user behavior has become crucial in securing the right players for each type of game. This study provides valuable insights for improving game development by measuring the electroencephalography (EEG) of game users and classifying the frequency of game usage. The multimodal mobile brain-body imaging (MOBI) dataset was employed for this study, and the frequency of game usage was categorized into ”often” and ”sometimes”. To achieve decent classification accuracy, a novel bimodal Transformer architecture featuring dedicated channels for the frontal (AF) and temporal (TP) lobes is introduced, wherein convolutional layers, self-attention mechanisms, and cross-attention mechanisms are integrated into a unified model. The model, designed to differentiate between AF and TP channels, exhibits functional differences between brain regions, allowing for a detailed analysis of inter-channel correlations. Evaluated through five-fold cross-validation (CV) and leave-one-subject-out cross-validation (LOSO CV), the proposed model demonstrates classification accuracies of 88.86% and 85.11%, respectively. By effectively classifying gameplay frequency, this methodology provides valuable insights for targeted game participation and contributes to strategic efforts to develop and design customized games for player acquisition.

## 1. Introduction

The introduction of video games has demonstrated a consistent upward trend, as evidenced by recent literature [1]. Furthermore, rapid advancements in computing technology have led to significant changes in the gaming industry [2], transforming games from mere forms of entertainment into significant tools in education, medicine, work management, and simulations [3]. In order to design a game that aligns with specific objectives, it is essential to objectively measure and analyze users’ cognitive actions during gameplay and reflect them in game design. Previously, game experiences were primarily assessed through subjective evaluations, such as questionnaires [4]. But efforts are being made to measure and analyze more objective biometric data [5]. In this paper, we aimed to conveniently acquire electroencephalography (EEG) data using a portable EEG device and enhance the accuracy of analysis using a deep learning model. EEG measured during gameplay can also be used to determine whether the game was “often” or “sometimes” used in the past. In other words, it is possible to classify whether a specific game is frequently used, serving as an objective indicator to determine interest in the game.

Recent research has specifically examined the effects of video games on mental health and cognitive development, revealing that video games have diverse impacts on cognitive abilities across various age groups. For example, it has been found that children who engage frequently in video gaming demonstrate improved cognitive abilities, including impulse control and working memory [6]. These findings are significant because they highlight the potential of video games as cognitive development tools during crucial developmental stages. Moreover, video games have shown promise in therapeutic contexts, particularly in improving attention spans among children diagnosed with attention deficit hyperactivity disorder (ADHD) [7]. This indicates that when video games are appropriately designed and utilized, they can serve as supplementary treatments for managing and potentially alleviating symptoms linked to ADHD. Research focusing on older adults suggested that video gaming improved cognitive functions in individuals in their 60s to levels similar to those in their 20s, and these effects lasted for up to six months after gameplay cessation [8]. Comparative studies of the brains of avid gamers, experienced in games such as “Starcraft” and “Warcraft”, compared with those with minimal gaming exposure, indicated heightened frontal lobe activity and enhanced learning capabilities in gamers during testing [9].

However, excessive video gaming can lead to addiction, significantly affecting health, academic performance, and social life. This is partly due to the role of dopamine, a neurotransmitter linked to pleasure and motivation [10]. Normally, dopamine boosts learning, memory, and motor skills [11]. Achievements trigger dopamine release, rewarding and motivating continued activity [12,13]. Conversely, excessive dopamine from activities like gaming can cause addiction. The striatum, crucial for learning and behavior, releases more dopamine during gaming, which may contribute to addictive patterns [14,15]. Furthermore, dopamine is implicated in various psychological disorders such as ADHD, autism, bipolar disorder, Parkinson’s disease, and schizophrenia, where an overactive dopamine system is common [16,17]. This highlights the complex effects of dopamine on behavior and psychological health. In light of these discussions, it becomes evident that video games embody a dual nature. This dichotomy reveals that, beyond temporary gratification, video games significantly enhance cognitive skills, foster creativity, and aid in the development of social competencies. Such positive outcomes hinge on the type of video games played and the frequency of engagement, thus challenging the conventional perception of gaming as inherently harmful. This nuanced understanding underscores the importance of a balanced approach to video gaming, recognizing its potential as a beneficial tool when used judiciously and in the appropriate contexts.

We utilized the globally acclaimed video game Minecraft, a sandbox game celebrated for its popularity and versatility, having sold over 100 million copies since its release [18]. Minecraft has served not only as a source of entertainment but also as a medium for creativity, learning, and social interaction [19]. Exploration in virtual environments like Minecraft enhance hippocampus-related memory, fostering children’s thinking and creativity [20]. The open-ended nature of the game also encourages innovative thinking, problem-solving, and collaborative learning, making it an increasingly popular educational tool worldwide [21]. Additionally, numerous studies have demonstrated that video gameplay can enhance task performance abilities in attention and perception tasks [22,23], including enhancing creativity, problem-solving skills, object detection abilities, visual-motor coordination, and spatial attention [24,25]. Therefore, classifying children based on gameplay frequency can provide insights into their cognitive, social, and emotional development [26]. Moreover, in order to further improve the gaming experience, assessing players’ cognitive states is crucial, for which EEG, measurable through wearable sensors, has been utilized. Alongside other neurophysiological tools, such as functional magnetic resonance imaging (fMRI) and magnetoencephalography (MEG), EEG plays a vital role in elucidating complex brain functions [27]. EEG stands out for high temporal resolution, offering significant advantages for precise and real-time monitoring of neural activities [28].

Our aim was to conduct a comprehensive analysis of gamers by distinguishing their gameplay frequency, utilizing a sophisticated deep learning model based on EEG. The proposed model separates and individually trains electrodes located in the frontal and temporal lobes using a convolutional neural network (CNN), recognizing that data from different brain regions offer unique insights. The Transformer module, employing self-attention and cross-attention mechanisms, extracts channel-wise global features for EEG data classification. This classification of EEG patterns between frequent and infrequent gamers is crucial for understanding user preferences and gameplay styles, informing game development and design, and exploring the potential of video games in cognitive therapy. This study suggests a new paradigm where regional EEG data-based user analysis helps games transcend mere entertainment, serving as tools for cognitive enhancement.

The expected contributions are as follows: (1) Our model advances neuroscientific approaches by proposing a new architecture that considers brain functions based on the location of EEG channels. The unique structure of this model treats channels as independent entities according to which brain regions they were acquitted from, enabling a nuanced analysis of cognitive and perceptual dynamics related to gaming activities. (2) The integration of convolutional layers, self-attention mechanisms, and cross-attention mechanisms within the region-dependent bimodal Transformer architecture represents a significant methodological advancement. This architecture facilitates the extraction of both local and global features and effectively utilizes the frontal and temporal lobe channels. Therefore, our approach not only brings about performance enhancements but also provides a clear direction for future research in applying complex neural network architectures to neuroscientific data.

The remaining paper is organized as follows. In Section 2, we describe the dataset used in the experiments, outline the preprocessing steps, and elucidate the proposed bimodal Transformer classification model. In Section 3, we present the classification results of the proposed model for the game frequency categories “sometimes” and “often”, and compare its accuracy with other models. The experimental results are discussed in Section 4. The conclusions are presented in Section 5.

## 2. Materials and Methods

### 2.1. EEG Dataset

**Figure 1:**
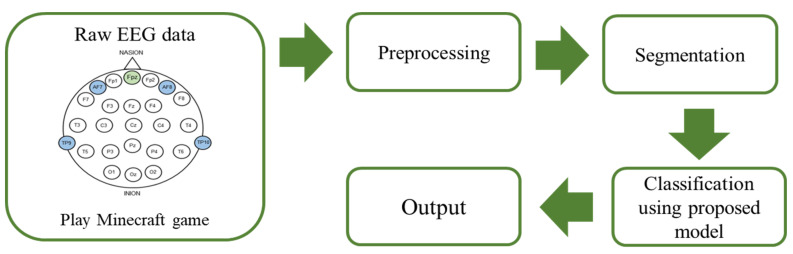
Block diagram for EEG-based gameplay frequency classification.

We used the multimodal mobile brain-body imaging (MOBI) dataset introduced by Ravindran et al. [29] in our study. This dataset includes data from two sessions: a 20 min Minecraft gameplay session and a 1 min resting interval. EEG data were collected using a Muse EEG headband, operating at a sampling frequency of 220 Hz. The Muse EEG headband features four active electrodes and employs a reference electrode positioned at the central forehead area, denoted as Fpz. EEG recordings were obtained from four channels using electrodes TP9, AF7, AF8, and TP10 at a sampling rate of 220 Hz. As illustrated in Figure 1, the even-numbered channels, AF8 and TP10, represent electrodes located in the right hemisphere of the brain, and the odd-numbered channels, AF7 and TP9, correspond to electrodes situated in the left hemisphere of the brain. AF7 and AF8 are situated in the frontal lobe, whereas TP9 and TP10 are located in the temporal lobe. Our focus in this study was on EEG data recorded during the 20 min Minecraft gameplay session.

Our research aimed to analyze the EEG signals captured during Minecraft gameplay sessions to develop an efficient classification scheme for individuals based on the distinct characteristics of their EEG signals. The primary goal was to explore potential changes in EEG patterns related to gameplay frequency. Specifically, we aimed to differentiate the frequency of Minecraft gameplay using the collected EEG data.

Table 1 presents the information of the EEG dataset for our experiments. The EEG data were obtained from 86 participants, consisting of 66 males and 20 females, aged between 6 and 16 years, with an average age of 8.8 and standard deviation of 2.40. Each participant’s EEG data were meticulously labeled according to their gameplay frequency for research purposes. Among these participants, 62 were categorized as “often” gamers, and the remaining 24 were classified as “sometimes” gamers.

### 2.2. Preprocessing

During the preprocessing stage, the raw EEG data collected via wireless headsets were inherently noisy, posing challenges to extracting meaningful information. EEG data can be affected by various artifacts, necessitating a preprocessing phase to enhance signal quality. We employed a fourth-order Butterworth band-pass filter with a passband of 1–50 Hz, a common denoising method, to address noise interference [30]. This filter effectively removed artifacts caused by biological movements. Following filtration, the data underwent downsampling. The signals were segmented into 2 s window frames, which were then inputted into the proposed model for further analysis. The length of each segment was calculated using the following formula:

(1) $$Length\;of\;each\;segment=\left\{seconds\right\}\times \{sampling\;rate\}$$

### 2.3. Bimodal Transformer Structure Featuring AF and TP Channels of EEG Data

**Figure 2:**
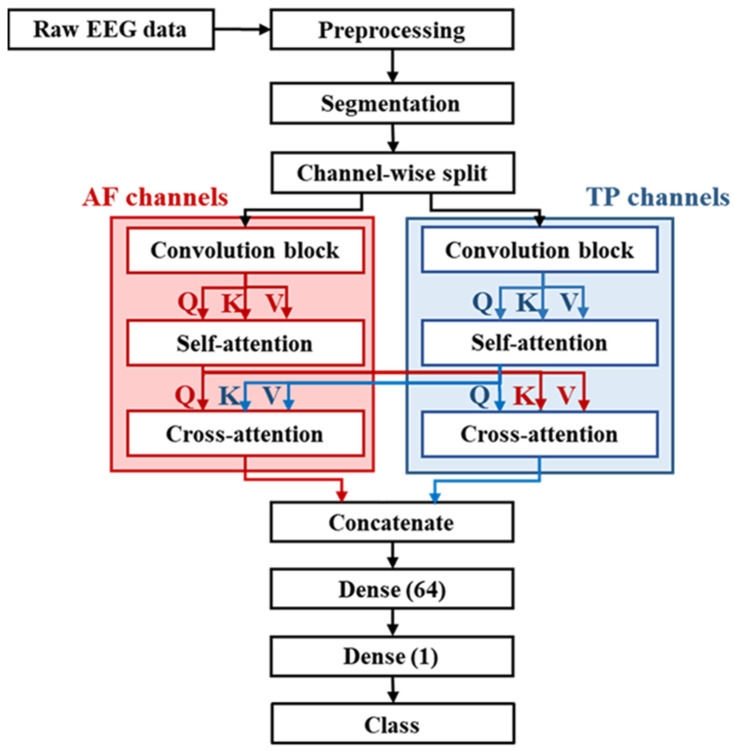
Overview of proposed bimodal Transformer architecture with regional EEG data.

The proposed model is a composite of convolution integrated with self-attention and cross-attention mechanisms, designed to leverage both local and global features in EEG data for accurate classification. Figure 2 illustrates the overall structure of our proposed model.

In the proposed methodology, convolution operations are applied to the AF and TP channels, with deliberate attention to both channel and temporal dimensions. The data from these distinct channels contain local information of the raw EEG data. The convolution mechanisms not only extract localized information but also reduce the resolution of the feature maps, streamlining the computational load for the subsequent self-attention layer. This approach also enables the effective extraction of global information from feature maps already rich with localized content. Following these operations, the cross-attention process extracts relational similarities between feature maps along the channel and temporal axes.

#### 2.3.1. AF and TP Channel Convolution

**Figure 3:**
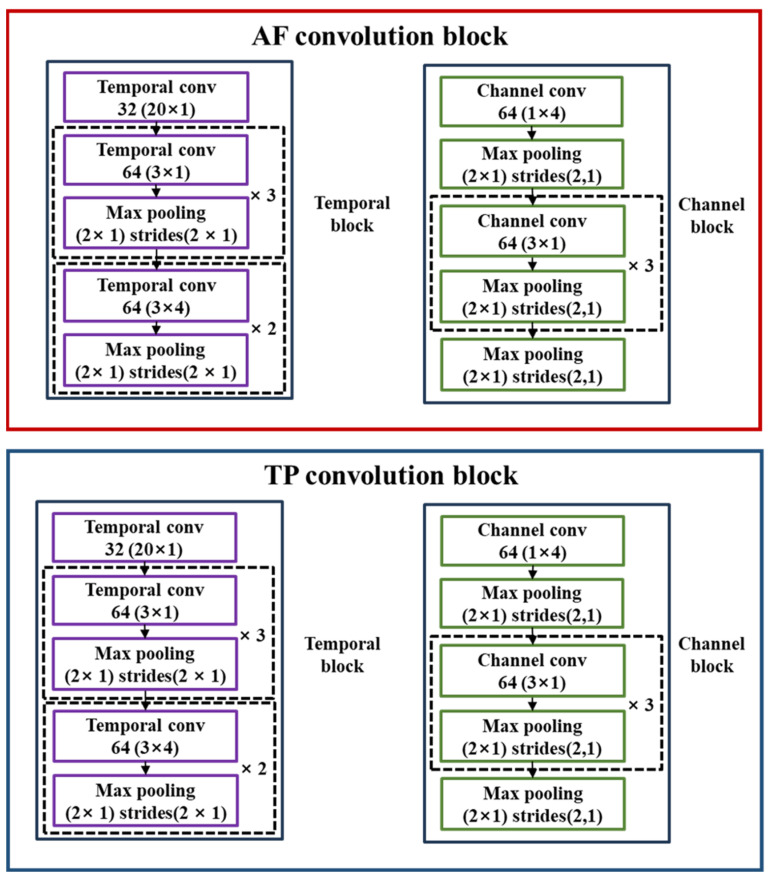
Convolution process distinguished by channel and temporal axes for AF and TP channels.

The initial processing involved preprocessing raw EEG signals and configuring the 1D signals from two TP channels and two AF channels into segments of 220 length, subsequently structuring the input shape into a 2D signal in the format of {length of each segment, number of channels} for model input. As a foundational step, we divided the channels associated with the frontal lobe (AF7 and AF8) and temporal lobe (TP9 and TP10) to facilitate individual convolution operations. Figure 3 illustrates the convolution procedures independently performed on the channel and temporal axes for each of the channels within the frontal and temporal lobes. The temporal block consists of 11 layers, comprising a combination of convolutional kernels and max pooling operations. Specifically, it includes 32 kernels of size 20 × 1 and 3 sets of 64 kernels of size 3 × 1 paired with 2 × 1 max pooling. Additionally, the block contains 2 sets of 64 kernels of size 3 × 4, each followed by a 2 × 1 max pooling operation. In contrast, the channel block comprises 9 layers structured around 64 kernels of size 1 × 4, followed by 2 × 1 max pooling. This setup is complemented by 3 sets of 64 channels of size 3 × 1, each accompanied by a 2 × 1 max pooling operation. The block concludes with a single 2 × 1 max pooling layer. This differentiation in processing highlights the complexity and region-specific functionalities of the brain. The frontal lobe, crucial for strategic thinking, problem-solving, decision-making, and emotional response regulation, is particularly relevant in gaming contexts requiring swift thinking and strategic actions [31]. Specifically, the right frontal cortex, a part of the frontal lobe, is known to be involved in regulating positive emotions, which are crucial for maintaining a positive gaming experience and making strategic decisions [32]. The left frontal lobe plays a crucial role in higher cognitive functions, including language processing, problem-solving, memory tasks, and the regulation of social behavior and decision-making [33]. Similarly, the temporal lobe, essential for auditory processing and memory retention, plays a vital role in navigating the complex dynamics of gaming [34]. The left temporal lobe is associated with the learning and retention of verbal materials, while the right temporal lobe is involved in the processing of non-verbal materials [35].

This approach acknowledges the distinct functionalities of the brain regions and their significance in gaming scenarios. By applying convolution operations separately on the channel and temporal axes, the model facilitates precise feature extraction tailored to each axis. This method enhances the ability of the model to extract relevant features, leveraging the unique contributions of both the frontal and temporal lobes to the gaming experience.

#### 2.3.2. Self-Attention and Cross-Attention Mechanisms

The self-attention block performs a 32-dimensional 1 × 1 convolution operation on the query, key, and value components. Subsequently, a multi-head attention mechanism with 4 heads is employed, followed by establishing a residual connection with the feature map from before the self-attention operation. The self-attention mechanism deployed in our experiments is characterized by 4 heads, 128 dimensions rate. For the TP axis, the self-attention process constructs query, key, and value vectors through dense layers from the TP input tensor. Subsequently, it performs a matrix multiplication between the query and the transposed key, applies a softmax function, and then carries out another matrix multiplication with the value. Similarly, the self-attention mechanism for the AF axis follows the same procedure with the AF input tensor.

By dissecting features derived from convolutions across different dimensions of the AF and TP channels, the model fosters interactions among these independent features, promoting a multimodal understanding rather than a singular interpretation. Consequently, the model output embodies both local and global inductive biases, fundamentally enhancing its generalization. This dual inductive bias arises from considering localized data while incorporating insights from a global perspective, significantly augmenting the generalization capabilities of the model. Additionally, the concurrent use of self-attention mechanisms and CNN leads to notable enhancements in memory efficiency.

After the self-attention block, a cross-attention block is implemented, mirroring the structure of the self-attention mechanism. In this setup, the queries from the AF and TP channels are crossed, enabling an interactive attention mechanism across various channel features. In our experiments, the cross-attention mechanism on the TP axis utilizes the output tensor from the TP’s self-attention, processed through dense layers, as the query. Meanwhile, the key and value are derived from the output tensor of the AF’s self-attention, also processed through dense layers. Conversely, the cross-attention mechanism on the AF axis employs the output tensor from the AF’s self-attention as the query and uses the processed output tensor from the TP’s self-attention for the key and value. This bidirectional cross-attention approach facilitates a nuanced exchange of information between the TP and AF axes, enhancing the model’s ability to capture complex interdependencies within the data. This cross-attention approach facilitates more comprehensive and nuanced feature integration between the distinct channel representations from different regions. This is particularly beneficial when collectively analyzing aspects from disparate domains. The essence of cross-attention lies in discerning correlations between different channels, as observed in this study between the AF channels in the frontal lobe and TP channels in the temporal lobe. By examining specific points within a channel, cross-attention infers correlations with the entirety of the channel, facilitating an understanding of the interactions of specific EEG patterns with the overall EEG pattern over time.

Therefore, this model demonstrates superior capability in recognizing and classifying complex EEG patterns more effectively. In addition to channel-specific understanding, cross-attention extracts correlation information to discern interactions and relationships between different channels, enabling the model to analyze more intricate EEG patterns. Therefore, through a more precise analysis facilitated by cross-attention, the model can derive more reliable results concerning EEG data.

## 3. Results

To evaluate the efficacy of our proposed approach, we employed five-fold cross-validation (CV) and leave-one-subject-out cross-validation (LOSO CV) methods. The five-fold CV involves dividing the entire dataset into five equal parts, using each part as test data and the remaining parts as training data, and repeating this process five times to derive the average results of each experiment. LOSO CV uses each subject’s data once as test data and the data from all other subjects as training data. Performance metrics, including accuracy, precision, recall, F1 score, and area under the curve (AUC) of the receiver operating characteristic (ROC) curve, were utilized to assess the efficacy of the model. The AUC serves as a widely accepted metric for test accuracy evaluation, with a value close to 1 indicating excellent performance. The terms TP, TN, FP, and FN represent true positive, true negative, false positive, and false negative, respectively. Accuracy, precision, recall, and F1 score are computed using Equations (2)–(5), respectively.

(2) $$Accuracy=\frac{TP+TN}{TP+TN+FP+FN}$$

(3) $$Precision=\frac{TP}{TP+FP}$$

(4) $$Recall=\frac{TP}{TP+FN}$$

(5) $$F1–score=2\times \frac{Precision\times Recall}{Precision+Recall}$$

### 3.1. Classification Results of the Proposed Model Comparing to Existing Models

This study utilizes the MOBI dataset [29] to evaluate our proposed model against evaluated models such as Ravindran et al. [29], EEGNet [36], BPR-STNet [37], and CoSleepNet [38] through the five-fold CV and LOSO CV methods. The comparative models are as follows:

- Ravindran et al. [29] is designed with separate spatial and temporal convolutions.
- EEGNet [36] features a compact and efficient CNN architecture with depthwise separable convolutions.
- BPR-STNet [37] is a neural architecture designed for the identification and classification of EEG data. It employs depthwise separable convolutions for efficient and effective feature extraction from spatiotemporal signals.
- CoSleepNet [38] introduces a cutting-edge hybrid architecture that combines CNN and long short-term memory (LSTM) networks, specifically designed for the automatic classification of EEG sleep stages.

The five-fold CV and LOSO CV results are presented in Table 2 and Table 3, comparing the proposed model with the evaluated models. Table 2 presents a comparative evaluation of the proposed model with other existing models using the average results of the five-fold cross-validation. This comprehensive comparison distinctly highlights the superior performance of our proposed model over other established methods. An analysis of the results revealed that our model achieved the highest classification accuracy of 88.86%. In contrast, the Ravindran et al. [29], EEGNet [36], BPR-STNet [37], and CoSleepNet [38] exhibited lower accuracies, achieving 81.83%, 76.60%, 79.32%, and 78.75%, respectively. This stark contrast highlights the superior predictive performance of our proposed model compared with the other models assessed. Collectively, the performance indicators corroborate the superior efficacy of the proposed model.

Table 3 illustrates a comparative evaluation between our proposed model and existing models using the LOSO CV method. In this approach, we utilized 86 participants, forming a training set from the data of 85 participants and using the remaining participant for testing. This procedure was meticulously repeated 86 times to ensure each participant was tested individually. The results from the LOSO CV demonstrate that our proposed model achieved an accuracy of 85.11%. The accuracy of our model shows an improved performance compared to other models. These findings underscore the superior predictive capability of our model, highlighting its effectiveness in accurately classifying EEG data. This demonstrates our proposed model’s superior predictive performance over the evaluated models, with overall performance metrics proving its effectiveness.

**Figure 4:**
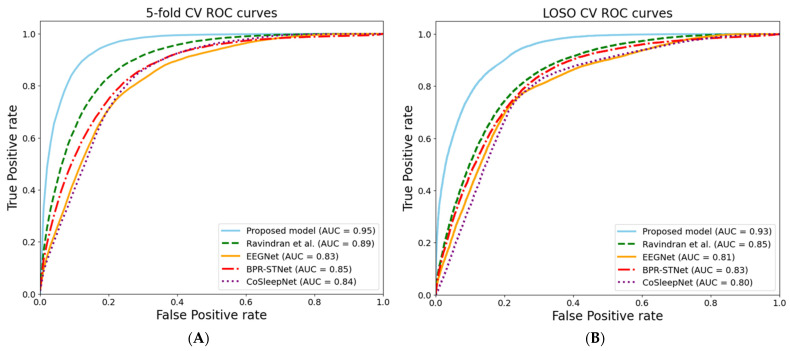
Comparison of ROC curve for the proposed model and the evaluated models: (**A**) Five-fold CV ROC curves. (**B**) LOSO CV ROC curves.

Figure 4 presents a comparison of the ROC curves for the proposed model and the evaluated models. The ROC curve serves as a graphical representation to evaluate the performance of binary classifier systems, depicting the trade-off between the true positive and false positive rates, thereby providing insights into the sensitivity and specificity of the model. Specifically, Figure 4A reveals an AUC score of 0.95 for the five-fold ROC curve of the proposed model, while Figure 4B shows an AUC of 0.93 for the LOSO CV ROC curve of the proposed model. These outcomes indicate superior performance, with scores closest to the ideal value of 1 among all comparative models. The proximity of these scores to the ideal value signifies the proposed model’s outstanding ability to differentiate between true positives and false positives. Higher AUC scores denote the enhanced capability of the model to accurately distinguish between classes, underscoring the exceptional performance of the proposed model.

#### Statistical Analysis according to the Classification Results

**Figure 5:**
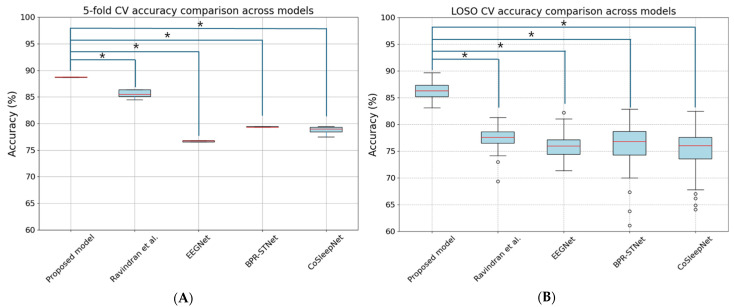
Mann–Whitney U Test results for accuracy comparisons between the proposed model and evaluated models: (**A**) Comparison of five-fold CV accuracy between the proposed model and other models. (**B**) Comparison of LOSO CV accuracy between the proposed model and other models. * (star) denotes statistically significant differences between the proposed model and other models (*p* < 0.01).

Figure 5 presents a statistical analysis of performance discrepancies between the proposed model and evaluated models, employing the non-parametric Mann–Whitney U test to assess the results from both the five-fold CV and LOSO CV.

For the five-fold CV presented in Figure 5A, we analyzed the accuracy per fold for our proposed model compared to Ravindran et al. [29], EEGNet [36], BPR-STNet [37], and CoSleepNet [38]. For the LOSO CV depicted in Figure 5B, we assessed the accuracy for each of the 86 participants, comparing our model against Ravindran et al. [29], EEGNet [36], BPR-STNet [37], and CoSleepNet [38].

Our findings indicate that the proposed model demonstrated statistically significant accuracy improvements over all evaluated models in both the five-fold CV and LOSO CV results (*p* < 0.01). The statistical significance, indicated by *p*-values less than 0.01, strongly validates the superior performance of our proposed model in terms of accuracy. This emphasizes its potential efficiency and reliability in classification tasks across different validation methodologies.

## 4. Discussion

In the modern landscape of video game research, integrating neurophysiological data, particularly EEG recordings, into user behavior analysis presents a groundbreaking approach to comprehending the intricacies of gaming engagement. This study leverages EEG data recorded during gaming sessions to classify the frequency of gameplay among users using the dataset amassed by Ravindran et al. [29]. Our investigation validates the premise that EEG data, providing rich insights into brain electrical activity, significantly enhance the precision of predicting gameplay frequency. This provides a robust framework for analyzing player behavior from a neuroscientific perspective.

This model achieved a notable improvement compared to the accuracy of other evaluated models. Previous studies have primarily conducted research using CNNs with EEG signals [39,40]. We have advanced this approach by proposing a new architecture that considers brain functions according to the position of the EEG channels. Our architecture uniquely combines convolution layers with self-attention and cross-attention mechanisms, significantly enhancing the predictive accuracy of the model. The significant improvement in predictive performance is attributed to the innovative architecture of our model, which seamlessly integrates convolutional layers with self-attention and cross-attention mechanisms. This fusion not only leverages the feature extraction capabilities of the CNN but also exploits the attention mechanisms’ dynamic feature prioritization, thereby enhancing the interpretability and predictive accuracy of the model.

Our model is structured to treat the AF and TP channels as separate entities, reflecting their distinct roles in cognitive processing and auditory functions, respectively. This design is crucial, considering the frontal lobe’s association with decision-making, problem-solving, and cognitive control, and the temporal lobe’s involvement in memory and auditory processing. This differentiation is particularly significant in gaming contexts where cognitive and perceptual processes are highly engaged. Moreover, empirical findings support the emphasis on these specific brain regions, indicating that skilled players demonstrate increased activity in these areas during intense gameplay, underscoring their pivotal role in gaming performance and engagement.

To implement this approach, our preprocessing pipeline segregates EEG data into AF and TP channels before independently applying convolution operations to each channel. This process facilitates feature extraction along both the channel and temporal axes, enabling a comprehensive analysis of EEG signals. Subsequently, the extracted features undergo synthesis through self-attention mechanisms, which assign greater weight to more pertinent features. This is followed by cross-attention mechanisms that assess the interplay between the frontal and temporal lobes. This sophisticated analysis framework accommodates the inherent individual variability in EEG data and significantly improves the generalization capabilities of the model across diverse user populations.

The implications of our findings extend beyond theoretical contributions to practical applications in game design and development. By elucidating the neurophysiological underpinnings of gameplay frequency, our model sets the stage for games to dynamically adapt to the cognitive and emotional states of the player, enhancing user engagement and personalizing the gaming experience. Furthermore, the insights gleaned could inform the design of neurofeedback games, where real-time brain activity influences game dynamics, providing a novel approach to sustaining player interest and engagement through personalized gameplay feedback.

### Limitations and Future Research

We designed an EEG classification model with high classification accuracy by considering the independent yet correlated characteristics of the frontal and temporal lobe channels in EEG signals. However, our study has several limitations. Firstly, for real-world applications in resource-constrained cases such as embedded systems, constructing a lightweight model is necessary. To this end, linear attention can be used as a substitute for the quadratic attention module, and it is necessary to use optimized kernel sizes depending on the classification application.

Additionally, the limited sample size we employed might restrict the applicability of our findings across diverse demographics and gaming habits. We plan to recruit a larger, more diverse participant pool in future studies, aiming to encompass a broader age range and various gaming experiences across different cultural backgrounds. This approach is crucial for validating the predictive reliability of our neuroscientific model across various populations, thereby gaining deeper insights into the relationship between gaming behavior and its neurophysiological correlates. Moreover, integrating techniques like variable-frequency complex demodulation (VFCDM) will allow for a more detailed exploration of the dynamic aspects of EEG signals [41]. This enhancement in our analytical precision will enable us to more distinctly identify varying brain states. Leveraging this, we plan to study cognitive states during gameplay across a diverse array of genres, from strategy and puzzles to action-adventure and role-playing games.

## 5. Conclusions

This study introduces a novel system for effectively classifying the frequency of video gameplay among users by leveraging EEG data and a deep learning bimodal Transformer model. Our approach involves the integration of convolution, self-attention, and cross-attention mechanisms into a bimodal Transformer model, specifically designed to analyze regional EEG data. We recognize the distinct roles that different brain regions play in cognitive processes and behavior patterns, hence treating the AF and TP lobe channels as separate domains to account for their functional differences. By analyzing the correlation results of these channels, our proposed model achieved impressive classification accuracies of 88.86% with five-fold CV and 85.11% with LOSO CV, surpassing the performance of previously reported models. Importantly, our research has broader applications beyond the specific games studied here and can be a valuable tool for assessing the educational effectiveness of games used for educational purposes.

## Figures and Tables

**Table 1:** Information of the EEG dataset for our experiments.

Information of the EEG Dataset for Our Experiments	
Number of participants	86
Number of males	66
Number of females	20
Age of participants	8.8 ± 2.40
Class	Played game often/sometimes
EEG device	Muse EEG headband
Recording EEG channel	TP9, AF7, AF8, TP10 (4 channel)
Sampling rate	220 Hz

**Table 2:** Five-fold cross-validation classification results for the proposed model compared to the evaluated models.

Model	Accuracy (%)	F1 Score (%)	Precision (%)	Recall (%)
Proposed model	88.86	85.81	86.03	87.67
Ravindran et al. [29]	81.83	78.81	77.48	80.64
EEGNet [36]	76.60	73.01	73.02	73.00
BPR-STNet [37]	79.32	73.53	74.41	73.91
CoSleepNet [38]	78.75	72.86	71.23	76.25

**Table 3:** Leave-one-subject-out cross-validation classification results for the proposed model compared to the evaluated models.

Model	Accuracy (%)	F1 Score (%)	Precision (%)	Recall (%)
Proposed model	85.11	83.29	81.75	84.89
Ravindran et al. [29]	77.63	74.63	74.01	75.27
EEGNet [36]	75.80	72.24	72.45	72.03
BPR-STNet [37]	76.16	72.75	72.71	72.78
CoSleepNet [38]	75.53	74.08	69.02	79.95

## Data Availability

The MOBI dataset is available at https://ieee-dataport.org/documents/multi-modal-mobile-brain-body-imaging-mobi-dataset-assaying-neural-and-head-movement (accessed on 22 December 2017).

## References

[1] Mathews C.L., Morrell H.E.R., Molle J.E. (2019). Video game addiction, ADHD symptomatology, and video game reinforcement. Am. J. Drug Alcohol Abuse.

[2] Anwar S.M., Saeed S.M.U., Majid M., Usman S., Mehmood C.A., Liu W. (2017). A game player expertise level classification system using electroencephalography (EEG). Appl. Sci..

[3] Olszewski A.E., Wolbrink T.A. (2017). Serious gaming in medical education: A proposed structured framework for game development. Simul. Healthc..

[4] Boyle E.A., Connolly T.M., Hainey T., Boyle J.M. (2012). Engagement in digital entertainment games: A systematic review. Comput. Hum. Behav..

[5] Hafeez T., Umar Saeed S.M., Arsalan A., Anwar S.M., Ashraf M.U., Alsubhi K. (2021). EEG in game user analysis: A framework for expertise classification during gameplay. PLoS ONE.

[6] Chaarani B., Ortigara J., Yuan D., Loso H., Potter A., Garavan H.P. (2022). Association of video gaming with cognitive performance among children. JAMA Netw. Open.

[7] Bioulac S., Lallemand S., Fabrigoule C., Thoumy A.L., Philip P., Bouvard M.P. (2014). Video game performances are preserved in ADHD children compared with controls. J. Atten. Disord..

[8] Anguera J.A., Boccanfuso J., Rintoul J.L., Al-Hashimi O., Faraji F., Janowich J., Kong E., Larraburo Y., Rolle C., Johnston E. (2013). Video game training enhances cognitive control in older adults. Nature.

[9] Kim Y.-H., Kang D.W., Kim D., Kim H.J., Sasaki Y., Watanabe T. (2015). Real-time strategy video game experience and visual perceptual learning. J. Neurosci..

[10] Wise R.A. (2004). Dopamine, learning and motivation. Nat. Rev. Neurosci..

[11] Weinstein A.M. (2010). Computer and Video Game Addiction—A Comparison between Game Users and Non-Game Users. Am. J. Drug Alcohol Abuse.

[12] Gebauer L., Kringelbach M.L., Vuust P. (2012). Ever-changing cycles of musical pleasure: The role of dopamine and anticipation. Psychomusicol. Music Mind Brain.

[13] Ferreiri L., Mas-Herrero E., Zatorre R.J., Ripollés P., Gomez-Andres A., Alicart H., Olivé G., Marco-Pallarés J., Antonijoan R.M., Valle A. (2019). Dopamine modulates the reward experiences elicited by music. Proc. Nat. Acad. Sci. USA.

[14] Dong G.H., Dong H., Wang M., Zhang J., Zhou W., Du X., Potenza M.N. (2021). Dorsal and ventral striatal functional connectivity shifts play a potential role in internet gaming disorder. Commun. Biol..

[15] Koepp M.J., Gunn R.N., Lawrence A.D., Cunningham V.J., Dagher A., Jones T., Grasby P.M. (1998). Evidence for striatal dopamine release during a video game. Nature.

[16] Previc F.H. (2007). Prenatal influences on brain dopamine and their relevance to the rising incidence of autism. Med. Hypotheses.

[17] Comings D.E., Wu S., Chiu C., Ring R.H., Gade R., Ahn C., Muhleman D. (1996). Polygenic inheritance of Tourette syndrome, stuttering, attention deficit hyperactivity, conduct, and oppositional defiant disorder: The additive and subtractive effect of the three dopaminergic genes—DRD2, DβH, and DAT1. Am. J. Med. Genet..

[18] Ali M., Mosa A.H., Al Machot F., Kyamakya K. EEG-based emotion recognition approach for e-healthcare applications. Proceedings of the 2016 Eighth International Conference on Ubiquitous and Future Networks (ICUFN).

[19] Davis K., Boss J.A., Meas P. (2018). Playing in the virtual sandbox: Students’ collaborative practices in Minecraft. Int. J. Game Based Learn..

[20] Fauzan N., Sophian Shminan A., James Anak Binit A.J.A. (2018). The effects of Minecraft videogame on creativity. Int. J. Eng. Technol..

[21] Boot W.R., Kramer A.F., Simons D.J., Fabiani M., Gratton G. (2008). The effects of video game playing on attention, memory, and executive control. Acta Psychol..

[22] Castel A.D., Pratt J., Drummond E. (2005). The effects of action video game experience on the time course of inhibition of return and the efficiency of visual search. Acta Psychol..

[23] Kühn S., Gleich T., Lorenz R.C., Lindenberger U., Gallinat J. (2014). Playing Super Mario induces structural brain plasticity: Gray matter changes resulting from training with a commercial video game. Mol. Psychiatry.

[24] Green C.S., Bavelier D. (2003). Action video game modifies visual selective attention. Nature.

[25] Clemenson G.D., Henningfield C.M., Stark C.E.L. (2019). Improving hippocampal memory through the experience of a rich Minecraft environment. Front. Behav. Neurosci..

[26] Andersen R., Rustad M. (2022). Using Minecraft as an educational tool for supporting collaboration as a 21st century skill. Comput. Educ. Open.

[27] Hasan M.N., Koo I. (2023). Mixed-input deep learning approach to sleep/wake state classification by using EEG signals. Diagnostics.

[28] Hondrou C., Caridakis G. (2012). Affective, natural interaction using EEG: Sensors, application and future directions. Artificial Intelligence: Theories and Applications, Proceedings of the 7: 7th Hellenic Conference on AI, SETN 2012, Lamia, Greece, 28–31 May 2012.

[29] Ravindran A.S., Mobiny A., Cruz-Garza J.G., Paek A., Kopteva A., Vidal J.L.C. (2019). Assaying neural activity of children during video game play in public spaces: A deep learning approach. J. Neural Eng..

[30] Heydari E., Shahbakhti M. Adaptive wavelet technique for EEG de-noising. Proceedings of the 8th Biomedical Engineering International Conference (BMEiCON).

[31] Barraclough D.J., Conroy M.L., Lee D. (2004). Prefrontal cortex and decision making in a mixed-strategy game. Nat. Neurosci..

[32] Veeranki Y.R., Kumar H., Ganapathy N., Natarajan B., Swaminathan R. (2021). A systematic review of sensing and differentiating dichotomous emotional states using audio-visual stimuli. IEEE Access.

[33] Chayer C., Freedman M. (2001). Frontal lobe functions. Curr. Neurol. Neurosci. Rep..

[34] Campitelli G., Gobet F., Head K., Buckley M., Parker A. (2007). Brain localization of memory chunks in chessplayers. Int. J. Neurosci..

[35] Lee T.M.C., Yip J.T.H., Jones-Gotman M. (2002). Memory deficits after resection from left or right anterior temporal lobe in humans: A meta-analytic review. Epilepsia.

[36] Lawhern V.J., Solon A.J., Waytowich N.R., Gordon S.M., Hung C.P., Lance B.J. (2018). EEGNet: A compact convolutional neural network for EEG-based brain–computer interfaces. J. Neural Eng..

[37] Lin L., Li P., Wang Q., Bai B., Cui R., Yu Z., Gao D., Zhang Y. (2024). An EEG-based cross-subject interpretable CNN for game player expertise level classification. Expert Syst. Appl..

[38] Efe E., Ozsen S. (2023). CoSleepNet: Automated sleep staging using a hybrid CNN-LSTM network on imbalanced EEG-EOG datasets. Biomed. Signal Process. Control.

[39] Aldawsari H., Al-Ahmadi S., Muhammad F. (2023). Optimizing 1D-CNN-Based Emotion Recognition Process through Channel and Feature Selection from EEG Signals. Diagnostics.

[40] Van Putten M.J., Olbrich S., Arns M. (2018). Predicting sex from brain rhythms with deep learning. Sci Rep..

[41] Veeranki Y.R., McNaboe R., Posada-Quintero H.F. (2023). EEG-Based Seizure Detection Using Variable-Frequency Complex Demodulation and Convolutional Neural Networks. Signals.

